# *N*′-(2,4-Dichloro­benzyl­idene)-2-methyl­benzohydrazide

**DOI:** 10.1107/S1600536810043710

**Published:** 2010-11-06

**Authors:** Chun-Bao Tang

**Affiliations:** a Department of Chemistry, Jiaying University, Meizhou 514015, People’s Republic of China

## Abstract

In the title hydrazone compound, C_15_H_12_Cl_2_N_2_O, the dihedral angle between the two benzene rings is 12.2 (2)°. In the crystal, mol­ecules are linked through inter­molecular N—H⋯O hydrogen bonds, forming forming *C*(4) chains propagating in [001].

## Related literature

For general background to hydrazones, see: Rasras *et al.* (2010 ▶); Pyta *et al.* (2010 ▶); Angelusiu *et al.* (2010 ▶). For the crystal structures of related compounds, see: Fun *et al.* (2008 ▶); Singh & Singh (2010 ▶); Ahmad *et al.* (2010 ▶); Tang (2010 ▶). For reference bond-length data, see: Allen *et al.* (1987 ▶).
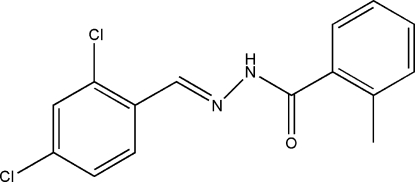


## Experimental

### 

#### Crystal data

C_15_H_12_Cl_2_N_2_O
*M* *_r_* = 307.17
Monoclinic, 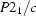

*a* = 7.563 (1) Å
*b* = 25.729 (2) Å
*c* = 8.174 (2) Å
β = 115.771 (2)°
*V* = 1432.4 (4) Å^3^
*Z* = 4
Mo *K*α radiation
μ = 0.45 mm^−1^
*T* = 298 K
0.15 × 0.13 × 0.10 mm

#### Data collection

Bruker SMART CCD area-detector diffractometer
Absorption correction: multi-scan (*SADABS*; Sheldrick, 1996 ▶) *T*_min_ = 0.936, *T*_max_ = 0.957
7436 measured reflections
3040 independent reflections
1529 reflections with *I* > 2σ(*I*)
*R*_int_ = 0.089

#### Refinement

*R*[*F* ^2^ > 2σ(*F* ^2^)] = 0.047
*wR*(*F* ^2^) = 0.097
*S* = 0.85
3040 reflections
185 parameters
1 restraint
H atoms treated by a mixture of independent and constrained refinement
Δρ_max_ = 0.22 e Å^−3^
Δρ_min_ = −0.18 e Å^−3^

### 

Data collection: *SMART* (Bruker, 2002 ▶); cell refinement: *SAINT* (Bruker, 2002 ▶); data reduction: *SAINT*; program(s) used to solve structure: *SHELXS97* (Sheldrick, 2008 ▶); program(s) used to refine structure: *SHELXL97* (Sheldrick, 2008 ▶); molecular graphics: *SHELXTL* (Sheldrick, 2008 ▶); software used to prepare material for publication: *SHELXL97*.

## Supplementary Material

Crystal structure: contains datablocks global, I. DOI: 10.1107/S1600536810043710/wn2414sup1.cif

Structure factors: contains datablocks I. DOI: 10.1107/S1600536810043710/wn2414Isup2.hkl

Additional supplementary materials:  crystallographic information; 3D view; checkCIF report

## Figures and Tables

**Table 1:** Hydrogen-bond geometry (Å, °)

*D*—H⋯*A*	*D*—H	H⋯*A*	*D*⋯*A*	*D*—H⋯*A*
N2—H2⋯O1^i^	0.90 (1)	2.03 (1)	2.892 (3)	159 (3)

Symmetry code: (i) 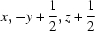.

**Fig. 1.**
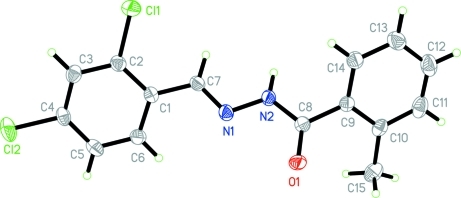
The molecular structure of the compound, showing the atom-numbering scheme. Displacement ellipsoids are drawn at the 30% probability level. H atoms are shown as spheres of arbitrary radius.

**Fig. 2.**
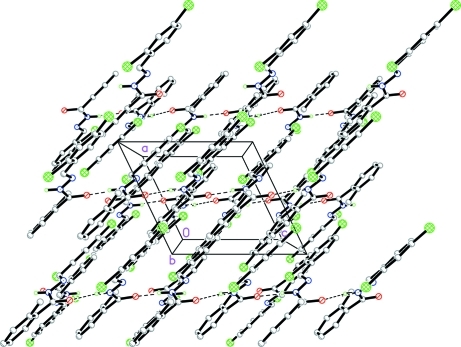
Molecular packing of the title compound, with hydrogen bonds shown as dashed lines.

## supplementary crystallographic information

### Comment

Hydrazone compounds have received much attention in biological and structural chemistry in the last few years (Rasras *et al.*, 2010; Pyta *et al.*, 2010; Angelusiu *et al.*, 2010; Fun *et al.*, 2008; Singh & Singh, 2010; Ahmad *et al.*, 2010). In the present paper, the author reports the crystal structure of the new title hydrazone compound (Fig. 1).

In the title molecule, the dihedral angle between the two benzene rings is 12.2 (2)°. The torsion angles C1—C7—N1—N2, C7—N1—N2—C8 and N1—N2—C8—C9 are 3.1 (2), 12.2 (2), and 3.0 (2)°, respectively. All the bond lengths have normal values (Allen *et al.*, 1987) and are comparable to those in the similar hydrazone compound reported recently (Tang, 2010).

In the crystal structure of the title compound, molecules are linked through intermolecular N—H···O hydrogen bonds (Table 1), forming chains along the *c* axis (Fig. 2).

### Experimental

2,4-Dichlorobenzaldehyde (0.1 mmol, 19.1 mg) and 2-methylbenzohydrazide (0.1 mmol, 15.0 mg) were dissolved in methanol (20 ml). The mixture was stirred at reflux for 10 min to give a clear colourless solution. Colourless block-shaped crystals of the compound were formed by slow evaporation of the solvent over several days.

### Refinement

Atom H2 was located in a difference Fourier map and refined isotropically, with the N—H distance restrained to 0.90 (1) Å [*U*_iso_(H) = 0.08 Å^2^]. Other H atoms were constrained to ideal geometries and refined as riding, with Csp^2^—H = 0.93 Å and C(methyl)—H = 0.96 Å; *U*_iso_(H) = k*U*_eq_(C), where k = 1.5 for methyl H and 1.2 for all other H atoms.

### Crystal data

**Table:**

C_15_H_12_Cl_2_N_2_O	*F*(000) = 632
*M**_r_* = 307.17	*D*_x_ = 1.424 Mg m^−^^3^
Monoclinic, *P*2_1_/*c*	Mo *K*α radiation, λ = 0.71073 Å
*a* = 7.563 (1) Å	Cell parameters from 1160 reflections
*b* = 25.729 (2) Å	θ = 2.7–24.3°
*c* = 8.174 (2) Å	µ = 0.45 mm^−^^1^
β = 115.771 (2)°	*T* = 298 K
*V* = 1432.4 (4) Å^3^	Block, colourless
*Z* = 4	0.15 × 0.13 × 0.10 mm

### Data collection

**Table:**

Bruker SMART CCD area-detector diffractometer	3040 independent reflections
Radiation source: fine-focus sealed tube	1529 reflections with *I* > 2σ(*I*)
graphite	*R*_int_ = 0.089
ω scans	θ_max_ = 27.0°, θ_min_ = 2.9°
Absorption correction: multi-scan (*SADABS*; Sheldrick, 1996)	*h* = −9→6
*T*_min_ = 0.936, *T*_max_ = 0.957	*k* = −32→28
7436 measured reflections	*l* = −10→10

### Refinement

**Table:**

Refinement on *F*^2^	Primary atom site location: structure-invariant direct methods
Least-squares matrix: full	Secondary atom site location: difference Fourier map
*R*[*F*^2^ > 2σ(*F*^2^)] = 0.047	Hydrogen site location: inferred from neighbouring sites
*wR*(*F*^2^) = 0.097	H atoms treated by a mixture of independent and constrained refinement
*S* = 0.85	*w* = 1/[σ^2^(*F*_o_^2^) + (0.0246*P*)^2^] where *P* = (*F*_o_^2^ + 2*F*_c_^2^)/3
3040 reflections	(Δ/σ)_max_ < 0.001
185 parameters	Δρ_max_ = 0.22 e Å^−^^3^
1 restraint	Δρ_min_ = −0.18 e Å^−^^3^

### Special details

**Table:**

Geometry. All e.s.d.'s (except the e.s.d. in the dihedral angle between two l.s. planes) are estimated using the full covariance matrix. The cell e.s.d.'s are taken into account individually in the estimation of e.s.d.'s in distances, angles and torsion angles; correlations between e.s.d.'s in cell parameters are only used when they are defined by crystal symmetry. An approximate (isotropic) treatment of cell e.s.d.'s is used for estimating e.s.d.'s involving l.s. planes.
Refinement. Refinement of *F*^2^ against ALL reflections. The weighted *R*-factor *wR* and goodness of fit *S* are based on *F*^2^, conventional *R*-factors *R* are based on *F*, with *F* set to zero for negative *F*^2^. The threshold expression of *F*^2^ > σ(*F*^2^) is used only for calculating *R*-factors(gt) *etc*. and is not relevant to the choice of reflections for refinement. *R*-factors based on *F*^2^ are statistically about twice as large as those based on *F*, and *R*-factors based on ALL data will be even larger.

### Fractional atomic coordinates and isotropic or equivalent isotropic displacement parameters (Å^2^)

**Table:**

	*x*	*y*	*z*	*U*_iso_*/*U*_eq_	
Cl1	0.30785 (12)	0.05472 (3)	0.66289 (10)	0.0762 (3)	
Cl2	−0.23133 (11)	0.02731 (3)	−0.01806 (10)	0.0694 (3)	
H2	0.424 (4)	0.2371 (10)	0.803 (2)	0.080*	
N1	0.3080 (3)	0.21618 (9)	0.5418 (2)	0.0400 (5)	
N2	0.4152 (3)	0.24626 (9)	0.6931 (3)	0.0412 (6)	
O1	0.4719 (3)	0.30767 (7)	0.5227 (2)	0.0506 (5)	
C1	0.1513 (3)	0.13548 (10)	0.4312 (3)	0.0354 (6)	
C2	0.1547 (3)	0.08202 (11)	0.4558 (3)	0.0411 (7)	
C3	0.0400 (4)	0.04854 (11)	0.3178 (3)	0.0455 (7)	
H3	0.0474	0.0128	0.3363	0.055*	
C4	−0.0840 (3)	0.06916 (12)	0.1541 (3)	0.0422 (7)	
C5	−0.0965 (4)	0.12162 (12)	0.1234 (3)	0.0473 (7)	
H5	−0.1832	0.1350	0.0114	0.057*	
C6	0.0214 (4)	0.15428 (11)	0.2613 (3)	0.0438 (7)	
H6	0.0142	0.1899	0.2403	0.053*	
C7	0.2715 (3)	0.17057 (11)	0.5775 (3)	0.0392 (7)	
H7	0.3215	0.1595	0.6976	0.047*	
C8	0.4929 (4)	0.29150 (10)	0.6711 (3)	0.0375 (6)	
C9	0.6141 (4)	0.31927 (10)	0.8446 (3)	0.0367 (6)	
C10	0.5972 (4)	0.37312 (11)	0.8577 (3)	0.0419 (7)	
C11	0.7197 (5)	0.39632 (12)	1.0223 (4)	0.0587 (8)	
H11	0.7096	0.4320	1.0353	0.070*	
C12	0.8536 (5)	0.36888 (15)	1.1652 (4)	0.0649 (9)	
H12	0.9344	0.3859	1.2720	0.078*	
C13	0.8691 (4)	0.31614 (14)	1.1512 (4)	0.0601 (9)	
H13	0.9602	0.2972	1.2482	0.072*	
C14	0.7481 (4)	0.29148 (11)	0.9917 (3)	0.0472 (7)	
H14	0.7564	0.2556	0.9827	0.057*	
C15	0.4557 (4)	0.40581 (11)	0.7048 (4)	0.0604 (9)	
H15A	0.4380	0.4386	0.7516	0.091*	
H15B	0.3317	0.3882	0.6475	0.091*	
H15C	0.5068	0.4115	0.6174	0.091*	

### Atomic displacement parameters (Å^2^)

**Table:**

	*U*^11^	*U*^22^	*U*^33^	*U*^12^	*U*^13^	*U*^23^
Cl1	0.0772 (6)	0.0571 (6)	0.0563 (5)	−0.0056 (4)	−0.0065 (4)	0.0130 (4)
Cl2	0.0518 (5)	0.0768 (6)	0.0616 (5)	−0.0099 (4)	0.0080 (4)	−0.0297 (4)
N1	0.0466 (14)	0.0428 (15)	0.0341 (12)	−0.0087 (12)	0.0208 (10)	−0.0055 (11)
N2	0.0559 (14)	0.0402 (15)	0.0316 (12)	−0.0128 (12)	0.0229 (12)	−0.0057 (11)
O1	0.0797 (14)	0.0437 (13)	0.0342 (10)	−0.0083 (10)	0.0301 (10)	0.0013 (8)
C1	0.0309 (14)	0.0418 (18)	0.0359 (15)	−0.0042 (13)	0.0169 (12)	−0.0042 (12)
C2	0.0340 (15)	0.0457 (19)	0.0390 (15)	−0.0025 (13)	0.0116 (12)	−0.0008 (13)
C3	0.0380 (16)	0.0406 (19)	0.0550 (17)	−0.0034 (13)	0.0175 (14)	−0.0038 (14)
C4	0.0309 (15)	0.051 (2)	0.0421 (16)	−0.0033 (13)	0.0134 (13)	−0.0131 (14)
C5	0.0403 (16)	0.062 (2)	0.0339 (15)	0.0094 (15)	0.0113 (13)	−0.0041 (14)
C6	0.0496 (17)	0.0418 (19)	0.0391 (16)	0.0049 (14)	0.0185 (14)	0.0018 (13)
C7	0.0411 (16)	0.0446 (19)	0.0303 (14)	−0.0041 (14)	0.0139 (12)	−0.0009 (13)
C8	0.0429 (16)	0.0371 (18)	0.0369 (15)	0.0020 (13)	0.0213 (13)	−0.0031 (13)
C9	0.0420 (16)	0.0399 (19)	0.0355 (15)	−0.0059 (13)	0.0236 (13)	−0.0013 (12)
C10	0.0492 (17)	0.0383 (19)	0.0520 (17)	−0.0061 (14)	0.0348 (15)	−0.0031 (14)
C11	0.076 (2)	0.050 (2)	0.066 (2)	−0.0191 (18)	0.046 (2)	−0.0198 (18)
C12	0.074 (2)	0.079 (3)	0.0475 (19)	−0.032 (2)	0.0317 (19)	−0.0183 (19)
C13	0.056 (2)	0.077 (3)	0.0438 (18)	−0.0152 (18)	0.0183 (16)	0.0042 (17)
C14	0.0507 (17)	0.0504 (19)	0.0431 (16)	−0.0092 (15)	0.0228 (14)	−0.0014 (15)
C15	0.065 (2)	0.043 (2)	0.079 (2)	0.0056 (16)	0.0376 (19)	0.0034 (17)

### Geometric parameters (Å, °)

**Table:**

Cl1—C2	1.731 (2)	C7—H7	0.9300
Cl2—C4	1.737 (2)	C8—C9	1.494 (3)
N1—C7	1.268 (3)	C9—C14	1.387 (3)
N1—N2	1.383 (3)	C9—C10	1.400 (3)
N2—C8	1.351 (3)	C10—C11	1.393 (4)
N2—H2	0.901 (10)	C10—C15	1.501 (4)
O1—C8	1.226 (3)	C11—C12	1.364 (4)
C1—C2	1.389 (3)	C11—H11	0.9300
C1—C6	1.393 (3)	C12—C13	1.371 (4)
C1—C7	1.458 (3)	C12—H12	0.9300
C2—C3	1.384 (3)	C13—C14	1.379 (3)
C3—C4	1.364 (3)	C13—H13	0.9300
C3—H3	0.9300	C14—H14	0.9300
C4—C5	1.368 (3)	C15—H15A	0.9600
C5—C6	1.377 (3)	C15—H15B	0.9600
C5—H5	0.9300	C15—H15C	0.9600
C6—H6	0.9300		
			
C7—N1—N2	114.4 (2)	O1—C8—C9	122.6 (2)
C8—N2—N1	118.9 (2)	N2—C8—C9	114.1 (2)
C8—N2—H2	120.6 (18)	C14—C9—C10	120.0 (2)
N1—N2—H2	120.3 (18)	C14—C9—C8	119.5 (2)
C2—C1—C6	116.7 (2)	C10—C9—C8	120.5 (2)
C2—C1—C7	121.9 (2)	C11—C10—C9	116.9 (3)
C6—C1—C7	121.4 (3)	C11—C10—C15	119.9 (3)
C3—C2—C1	122.2 (2)	C9—C10—C15	123.2 (2)
C3—C2—Cl1	117.4 (2)	C12—C11—C10	122.7 (3)
C1—C2—Cl1	120.39 (19)	C12—C11—H11	118.7
C4—C3—C2	118.5 (3)	C10—C11—H11	118.7
C4—C3—H3	120.7	C11—C12—C13	120.0 (3)
C2—C3—H3	120.7	C11—C12—H12	120.0
C3—C4—C5	121.8 (2)	C13—C12—H12	120.0
C3—C4—Cl2	118.6 (2)	C12—C13—C14	119.2 (3)
C5—C4—Cl2	119.6 (2)	C12—C13—H13	120.4
C4—C5—C6	118.9 (2)	C14—C13—H13	120.4
C4—C5—H5	120.5	C13—C14—C9	121.1 (3)
C6—C5—H5	120.5	C13—C14—H14	119.4
C5—C6—C1	121.9 (3)	C9—C14—H14	119.4
C5—C6—H6	119.0	C10—C15—H15A	109.5
C1—C6—H6	119.0	C10—C15—H15B	109.5
N1—C7—C1	120.3 (2)	H15A—C15—H15B	109.5
N1—C7—H7	119.9	C10—C15—H15C	109.5
C1—C7—H7	119.9	H15A—C15—H15C	109.5
O1—C8—N2	123.2 (2)	H15B—C15—H15C	109.5

### Hydrogen-bond geometry (Å, °)

**Table:**

*D*—H···*A*	*D*—H	H···*A*	*D*···*A*	*D*—H···*A*
N2—H2···O1^i^	0.90 (1)	2.03 (1)	2.892 (3)	159 (3)

Symmetry codes: (i) *x*, −*y*+1/2, *z*+1/2.

## References

[bb1] Ahmad, T., Zia-ur-Rehman, M., Siddiqui, H. L., Mahmud, S. & Parvez, M. (2010). *Acta Cryst.* E**66**, o976. 10.1107/S1600536810011244 PMC2983976 21580774

[bb2] Allen, F. H., Kennard, O., Watson, D. G., Brammer, L., Orpen, A. G. & Taylor, R. (1987). *J. Chem. Soc. Perkin Trans. 2*, pp. S1–19.

[bb3] Angelusiu, M. V., Barbuceanu, S. F., Draghici, C. & Almajan, G. L. (2010). *Eur. J. Med. Chem.* **45**, 2055–2062. 10.1016/j.ejmech.2010.01.033 20133023

[bb4] Bruker (2002). *SMART* and *SAINT* Bruker AXS Inc., Madison, Wisconsin, USA.

[bb5] Fun, H.-K., Sujith, K. V., Patil, P. S., Kalluraya, B. & Chantrapromma, S. (2008). *Acta Cryst.* E**64**, o1961–o1962. 10.1107/S1600536808029218 PMC2959329 21201163

[bb6] Pyta, K., Przybylski, P., Huczynski, A., Hoser, A., Wozniak, K., Schilf, W., Kamienski, B., Grech, E. & Brzezinski, B. (2010). *J. Mol. Struct.* **970**, 147–154.

[bb7] Rasras, A. J. M., Al-Tel, T. H., Al-Aboudi, A. F. & Al-Qawasmeh, R. A. (2010). *Eur. J. Med. Chem.* **45**, 2307–2313. 10.1016/j.ejmech.2010.02.006 20181416

[bb8] Sheldrick, G. M. (1996). *SADABS* University of Göttingen, Germany.

[bb9] Sheldrick, G. M. (2008). *Acta Cryst.* A**64**, 112–122. 10.1107/S0108767307043930 18156677

[bb10] Singh, V. P. & Singh, S. (2010). *Acta Cryst.* E**66**, o1172. 10.1107/S1600536810010937 PMC2979270 21579213

[bb11] Tang, C.-B. (2010). *Acta Cryst.* E**66**, o2482. 10.1107/S1600536810035063 PMC2983165 21587483

